# Characterization of Novel and Uncharacterized p53 SNPs in the Chinese Population – Intron 2 SNP Co-Segregates with the Common Codon 72 Polymorphism

**DOI:** 10.1371/journal.pone.0015320

**Published:** 2011-01-10

**Authors:** Beng Hooi Phang, Hui Wan Chua, Huihua Li, Yeh Ching Linn, Kanaga Sabapathy

**Affiliations:** 1 Division of Cellular and Molecular Research, Humphrey Oei Institute of Cancer Research, National Cancer Centre, Singapore, Singapore; 2 Division of Clinical Trials and Epidemiological Sciences, Humphrey Oei Institute of Cancer Research, National Cancer Centre, Singapore, Singapore; 3 Department of Hematology, Singapore General Hospital, Singapore, Singapore; 4 Department of Biochemistry, Yong Loo Lin School of Medicine, National University of Singapore, Singapore, Singapore; 5 Cancer and Stem Cell Biology Program, Duke-National University of Singapore Graduate Medical School, Singapore, Singapore; Duke-NUS Graduate Medical School, Singapore

## Abstract

Multiple single nucleotide polymorphisms (SNPs) have been identified in the tumor suppressor gene *p53*, though the relevance of many of them is unclear. Some of them are also differentially distributed in various ethnic populations, suggesting selective functionality. We have therefore sequenced all exons and flanking regions of *p53* from the Singaporean Chinese population and report here the characterization of some novel and uncharacterized SNPs - four in intron 1 (nucleotide positions 8759/10361/10506/11130), three in intron 3 (11968/11969/11974) and two in the 3′UTR (19168/19514). Allelic frequencies were determined for all these and some known SNPs, and were compared in a limited scale to leukemia and lung cancer patient samples. Intron 2 (11827) and 7 (14181/14201) SNPs were found to have a high minor allele frequency of between 26–47%, in contrast to the lower frequencies found in the US population, but similar in trend to the codon 72 polymorphism (SNP12139) that shows a distribution pattern correlative with latitude. Several of the SNPs were linked, such as those in introns 1, 3 and 7. Most interestingly, we noticed the co-segregation of the intron 2 and the codon 72 SNPs, the latter which has been shown to be expressed in an allele-specific manner, suggesting possible regulatory cross-talk. Association analysis indicated that the T/G alleles in both the co-segregating intron 7 SNPs and a 4tagSNP haplotype was strongly associated increased susceptibility to lung cancer in non-smoker females [OR: 1.97 (1.32, 3.394)]. These data together demonstrate high SNP diversity in *p53* gene between different populations, highlighting ethnicity-based differences, and their association with cancer risk.

## Introduction

The tumor-suppressor gene *p53* is highly mutated up to 50% in all human cancer types [1], making it the most genetically targeted gene involved in carcinogeneis. Mutations often lead to loss of the tumor-suppressive functions of p53 - especially the transcription-dependent functions, highlighted by the enormous hot-spot mutations in the DNA-binding domain - thereby leading to uncontrolled cellular growth [1]–[3]. Besides mutations, p53 is thought also to be functionally inactivated through deregulated upstream and downstream regulators [1]–[3]. A case in point is the overexpression of its negative regulator MDM2, which leads to the rapid degradation of p53 [4]–[6]. In addition, p53 can also be functionally incapacitated by altered activation mechanisms involving post-translational modifications [7], [8]. p53 therefore is not mutated in these cases but is unable to perform its functions optimally due to lack of activation, as has also been noted in the case of neuroblastomas where it is often sequestered in the cytoplasm [9].

Being the most important tumor suppressor gene, the association of *p53* status with cancer risk has always been a captivating area of work. As mutations do not lead to elevated non-familial cancer risk in population-based studies (except in the Li-Fraumeni syndrome families with germ-line *p53* mutations), the role of single nucleotide polymorphisms (SNPs) in *p53* and their association with cancer risk has been studied extensively. It is noteworthy that there are about 90 SNPs reported in the *p53* gene, of which only six are in coding exons [10]. The relevance of the majority of the intronic SNPs is at present unclear. However, a few of the intronic SNPs, in particular, those in intron 3 (SNP11951) and intron 6 (SNP13494 and 13964) has been associated with cancer predisposition [11]–[14]. Nonetheless, the functional relevance of the different intronic SNPs in affecting cancer risk is not well understood. Moreover, most of the other intronic SNPs have not been well characterized, or little information including allelic frequency in various populations is available to understand their significance. Of the exonic SNPs, only two, in exon 4, are non-synonymous SNPs leading to altered amino-acids (SNP12063 encoding for codon 47 and SNP12139 coding for codon 72) [15]. Both these polymorphisms have been suggested to affect p53 function to varying extents, though the relevance of the codon 72 polymorphism has been extensively studied over the last 2 decades [15], [16]. The codon 72 polymorphism has been controversially associated with cancer risk in many cases. Essentially, both the G (giving rise to an arginine amino-acid) or C (giving rise to a proline amino-acid) alleles have been found to be associated with various types of cancer risks, though such associations have not been consistently noted in different populations [17]–[26]. Interestingly, there is a clear trend in the distribution of these polymorphic alleles, and a correlation exists between the presence of the G allele and distance away from the Equator [27]. Therefore, populations such as the Africans who live closer to the Equator tend to have a larger proportion of C-allele carriers compared to the Northern Europeans who are predominantly the G-allele carriers [27]. Thus, ethnicity has been suggested to be a critical factor in determining the effects of these different alleles on cancer predisposition [18], [20], [23], underlining the need for understanding the allelic distribution in various populations.

Functionally, the different codon 72 polymorphic variants have been shown to affect p53 function differentially. The arginine-variant was found to have a higher ability to induce mitchondrial-mediated apoptosis [28]. In contrast, the proline-variant was found to have a better capacity to induce DNA-repair and cell-cycle arrest, by virtue of differential activation of p53-target genes [29], [30]. Moreover, these variants have differing affinities to protein partners such as iASPP and MDM2 [28], [31], thereby suggesting that the structural changes may influence the function of these p53 forms.

We have previously reported that there is an allele-specific expression of the codon72-polymorphic variant p53 forms, both in heterozygous normal and cancer cohorts [17]. In normal healthy individuals, the proline-variant was preferentially expressed in contrast to the preferential expression of the arginine-variant in the breast cancer heterozygote cases [17]. How this allele-specific expression is regulated is unclear. Thus, in an attempt to investigate if there are other regulatory regions that may have relevance to this allele-specific expression, we embarked on sequencing the *p53* gene locus to identify polymorphisms that may be linked to the codon 72 polymorphism, as well as to identify novel SNPs and to characterize the frequency of some of the SNPs in the Chinese population, since population-based variations in allele frequencies have been demonstrated for p53 SNPs [27]. Here we report the characterization of several novel and already known SNPs in the Chinese population, and note an ethnicity-based association of some SNP alleles that are predominant in the Chinese population compared to the US population (which comprises of 24 African Americans, 24 Asian Americans, 24 European Americans, 12 Hispanic American, 6 Native Americans). Moreover, we show the complete linkage of an intron 2 SNP with the codon 72 polymorphism, suggesting that this SNP pair may have relevance in determining the preferential expression of the codon 72 polymorphic variants. Finally, we also noted association of the intron 7 SNPs with cancer risk, both alone or in the haplotype analysis in a limited scale study of female non-smoker lung-cancer samples. Detailed results are presented.

## Results

### Identification of novel SNPs in p53 and their allelic frequencies

We sequenced the regions covering all the exons and their flanking regions of the *p53* gene of about 11 kb from genomic DNA of healthy volunteers, from intron 1 (from nucleotide position 8634) to the 3′UTR region (19715), and identified several intronic SNPs that have not been previously reported or have been reported but not characterized. These included four in intron 1 (at positions 8759, 10361, 10506 and 11130), three in intron 3 (11968, 11969 and 11974) and two in the 3′UTR (19168 and 19514). Comparison with HapMap data indicated that of these, one SNP in intron 1 (10506) and all three in intron 3 (11968, 11969 and 11974) have not been previously reported. Table 1 shows the SNPs in *p53* that we identified, together with some of the others that are already known and is the subject of this study. The SNP alleles specifically characterized in this study are as follows: in intron 1 - 8759T/C, 10361G/A, 10506T/C and 11130A/G; intron 3 – 11968G/A, 11969G/C and 11974G/A; 3′UTR – 19168G/A and 19514G/A.

**Table 1:** List of SNPs analyzed among Chinese healthy controls.

SNP(Intron/exon …nucleotide position)	Minor allele	Major allele	Minor allele frequency (95% CI)	Minor allele frequency from HapMap(US population)	Minor allele frequency from HapMap(Chinese population)
In.1….8759	C	T	0.292 (0.214, 0.385)	0.19	Not reported
In.1….10361	A	G	0.311 (0.231, 0.404)	0.29	Not reported
In.1….10506	C	T	0.274 (0.198, 0.366)	Not reported	Not reported
In.1. …11130	A	G	0.292 (0.214, 0.385)	0.17	Not reported
In.2….11827	C	G	0.470 (0.355, 0.589)	0.25	Not reported
In.3….11968	A	G	0.009 (0.002, 0.051)	Not reported	Not reported
In.3….11969	C	G	0.009 (0.002, 0.051)	Not reported	Not reported
In.3….11974	A	G	0.009 (0.002, 0.051)	Not reported	Not reported
In.3….11992	A	C	0.029 (0.010, 0.082)	0.05	Not reported
Ex.4….12139	C	G	0.470 (0.355, 0.589)	0.35	0.50
In.6….13494	A	G	0.028 (0.01, 0.079)	0.06	0.034
In.7….14181	T	C	0.264 (0.189, 0.355)	0.16	Not reported
In.7….14201	G	T	0.264 (0.189, 0.355)	0.083	0.39
3′UTR..19168	A	G	0.117 (0.067, 0.197)	0.10	0.044
3′UTR..19514	A	G	0.052 (0.022, 0.116)	0.04	Not reported

The allelic frequencies of these novel and some of the other known SNPs were determined using samples from healthy Chinese population [17] (Table 1). All SNPs in controls were in Hardy-Weinberg Equilibrium. Minor allele frequencies (MAF) of each SNP, together with 95% CI are listed in Table 1. Among these 15 SNPs analyzed (including the novel ones), three SNPs at intron 3 (11968, 11969, 11974) had MAF of less than 1%. Of the remaining 12 SNPs, the MAF ranges from 2.8% (SNP 13494) to 47.0% (11827 and 12139 [codon 72]). Of note, several SNPs in introns 1, 2 and 7 had MAF between 26–46%, in contrast to lower frequencies reported in the US population [10], suggesting that these SNPs may have important functional relevance in p53 biology in a population-dependent manner.

### Co-segregating SNPs

**Figure 1:**
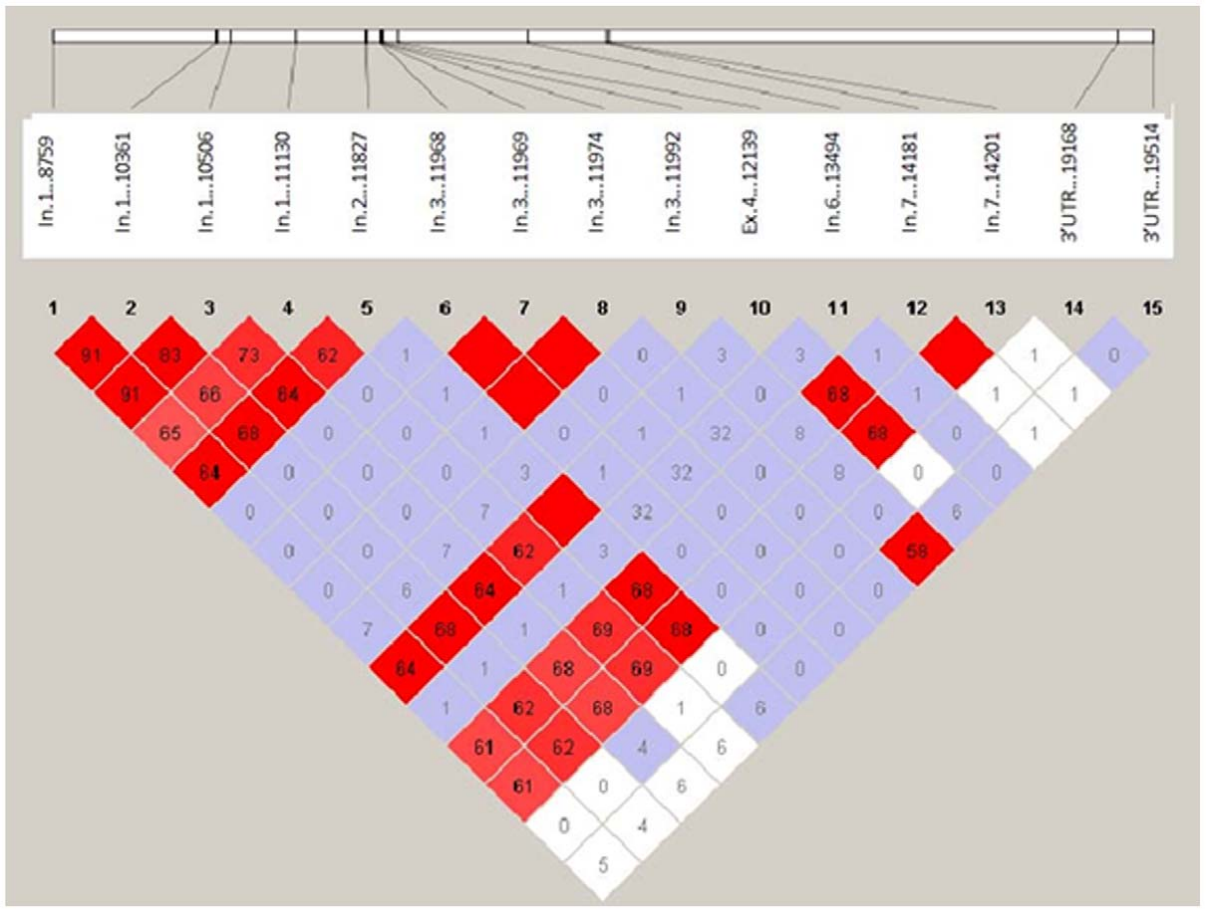
Graph showing pairwise r^2^ linkage disequilibrium (LD). Squares in red with no numerical indicates complete LD between markers.

Of the newly characterized SNPs, several of them co-segregated. The pattern of pairwise linkage disequilibrium (LD) between the various SNPs among controls are shown in Figure 1. For example, the intron 1 SNPs 8759T/C co-segregated with SNP 10361G/A (1602 nucleotides apart) and SNP 10506T/C (1747 nucleotides apart) (Figure 1). The SNPs at position 8759, 10361 and 10506 in intron 1 were in strong LD to each other with r^2^ greater than 0.8. Similarly, the three SNPs in intron 3 including SNP11968, 11969 and 11974 were in complete LD to each other (all r^2^ = 1) (Figure 1).

We also identified a linkage between previously noted SNPs, such as that in intron 7 (32). The two SNPs in intron 7 (14181 and 14201) were also in complete LD (r^2^ equal to 1). Moreover, we also found that the SNP in intron 2 (11827) co-segregated with the common codon 72 polymorphic SNP12139 in exon 4 (Figure 1). A noteworthy point is that the intervening coding regions, which contain the SNPs in intron 3, were not linked with these alleles. Moreover, the intron 7 SNPs have some level of LD with the intron 1 and 2 SNPs (r^2^>0.6), though the intervening SNP13494 site in intron 6 was not linked with them, highlighting gene rearrangements in the *p53* gene. Thus, the data analyses have uncovered previously unnoticed LD associations, especially between the intron 2 SNP11827 and the codon 72 SNP12139, the latter which has been associated with cancer predisposition [19], [21], [22], [25], [26].

### SNPs and association with cancer susceptibility

In order to determine if the newly identified SNPs have any association with cancer susceptibility, we analyzed the distribution of some of the alleles of the SNPs in healthy normal donors, and in a pilot study with a small group of leukemia patients or in a group of non-smoker female lung cancer patients, which have been used in our previous p53 association studies. The leukemia group analyzed consisted of up to 44 Chinese patients whose DNA were previously used for analysis of the effect of p53 codon 72 polymorphism (in exon 4), which revealed no significant association of this polymorphism on leukemia susceptibility [33]. The lung cancer cohort consisted of up to 79 patient samples, also utilized in a previous study analyzing the effects of p53 codon 72 polymorphism and MDM SNP309, which found no significant association of the codon 72 SNP with lung cancer susceptibility in female [34].

Association tests of each SNP showed that none of the analyzed SNPs were associated with leukemia risk (Table 2), while 2 of 6 SNPs (14181 and 14201) investigated were found to have significant association with increased lung cancer risk (Table 3).

**Table 2:** Risk estimates (OR* and 95% CI#) for leukemia for individual SNP.

Intron/Exon…SNP	Model	Controls		Cases		OR (95% CI)	GlobalP	P trend
		n	%	n	%			
In.3…11968	Genotype						1.000	0.349
	AA	0	0.0	0	0.0			
	AG	1	1.9	0	0.0	0.376 (0.015, 9.465)		
	GG	52	98.1	44	100.0	-		
	Allele						1.000	
	A	1	0.9	0	0.0	0.380 (0.015, 9.447)		
	G	105	99.1	88	100.0	-		
In.3…11969	Genotype						1.000	0.349
	CC	0	0.0	0	0.0			
	CG	1	1.9	0	0.0	0.376 (0.015, 9.465)		
	GG	52	98.1	44	100.0	-		
	Allele						1.000	
	C	1	0.9	0	0.0	0.380 (0.015, 9.447)		
	G	105	99.1	88	100.0	-		
In.3…11974	Genotype						1.000	0.349
	AA	0	0.0	0	0.0			
	AG	1	1.9	0	0.0	0.376 (0.015, 9.465)		
	GG	52	98.1	44	100.0	-		
	Allele						1.000	
	A	1	0.9	0	0.0	0.380 (0.015, 9.447)		
	G	105	99.1	88	100.0	-		
In.3…11992	Genotype						1.000	0.750
	AA	0	0.0	0	0.0			
	AC	3	5.8	2	4.5	0.742 (0.119, 4.651)		
	CC	49	94.2	42	95.5	-		
	Allele						1.000	
	A	3	2.9	2	2.3	0.748 (0.122, 4.579)		
	C	101	97.1	86	97.7	-		
In.6…13494	Genotype						0.675	0.570
	AA	0	0.0	0	0.0			
	AG	3	5.7	3	8.8	1.613 (0.306, 8.498)		
	GG	50	94.3	31	91.2	-		
	Allele						0.680	
	A	3	2.8	3	4.4	1.585 (0.310, 8.089)		
	G	103	97.2	65	95.6	-		
In.7…14181	Genotype						0.641	0.435
	TT	4	7.5	2	8.7	1.450 (0.230, 9.160)		
	TC	20	37.7	11	47.8	1.595 (0.570, 4.461)		
	CC	29	54.7	10	43.5	-		
	Allele						0.440	
	T	28	26.4	15	32.6	1.348 (0.635, 2.861)		
	C	78	73.6	31	67.4	-		
In.7…14201	Genotype						0.641	0.435
	GG	4	7.5	2	8.7	1.450 (0.230, 9.160)		
	GT	20	37.7	11	47.8	1.595 (0.570, 4.461)		
	TT	29	54.7	10	43.5	-		
	Allele						0.440	
	G	28	26.4	15	32.6	1.348 (0.635, 2.861)		
	T	78	73.6	31	67.4	-		
3′UTR…19168	Genotype						0.161	0.135
	AA	0	0.0	0	0.0			
	AG	11	23.4	4	10.8	0.397 (0.115, 1.368)		
	GG	36	76.6	33	89.2	-		
	Allele						0.182	
	A	11	11.7	4	5.4	0.432 (0.131, 1.414)		
	G	83	88.3	70	94.6	-		

*: OR – Odds ratio.

# : CI - confidence interval.

**Table 3:** Risk estimates (OR and 95% CI) for lung cancer for individual SNP.

SNP	Model	Controls		Cases		OR (95% CI)	Global P	P trend
		n	%	n	%			
In.2…11827	Genotype						0.782	0.621
	CC	15	28.3	16	22.5	0.782 (0.274, 2.234)		
	CG	27	50.9	40	56.3	1.086 (0.434, 2.722)		
	GG	11	20.7	15	21.1	-		
	Allele						0.700	
	C	57	53.8	72	50.7	1.131 (0.683, 1.872)		
	G	49	46.2	70	49.3	-		
In.3…11992	Genotype						0.119	0.070
	AA	0	0.0	1		4.183 (0.166, 105.695)		
	AC	3	5.8	6		2.800 (0.655, 11.963)		
	CC	49	94.2	35		-		
	Allele						0.066	
	A	3	2.9	8		3.544 (0.910, 13.806)		
	C	101	97.1	76		-		
Ex.4…12139	Genotype						0.782	0.621
	CC	15	28.3	16	22.5	0.782 (0.274, 2.234)		
	CG	27	50.9	40	56.3	1.086 (0.434, 2.722)		
	GG	11	20.7	15	21.1	-		
	Allele						0.700	
	C	57	53.8	72	50.7	1.131 (0.683, 1.872)		
	G	49	46.2	70	49.3	-		
In.6…13494	Genotype						0.312	0.185
	AA	0	0.0	0		-		
	AG	3	5.7	1		0.238 (0.024, 2.356)		
	GG	50	94.3	70		-		
	Allele						0.316	
	A	3	2.8	1		0.243 (0.0250, 2.374)		
	G	103	97.2	141		-		
In.7…14181	Genotype						**0.018**	**0.011**
	TT	4	7.5	8		2.900 (0.768, 10.949)		
	TC	20	37.7	41		**2.973 (1.361, 6.492)**		
	CC	29	54.7	20		-		
	Allele						**0.021**	
	T	28	26.4	57		**1.960 (1.132, 3.394)**		
	C	78	73.6	81		-		
In.7…14201	Genotype						**0.014**	**0.010**
	GG	4	7.5	8		2.900 (0.768, 10.949)		
	GT	20	37.7	42		**3.045 (1.396, 6.641)**		
	TT	29	54.4	20		-		
	Allele						**0.016**	
	G	28	26.4	58		**1.970 (1.140, 3.406)**		
	T	78	73.6	82		-		

Based on the LD pattern among the 6 SNPs investigated in lung cancers with MAF at least 1%, 4 tagSNPs (SNPs 11827, 11992, 13494 and 14201) were selected for further haplotype analyses. The two most common haplotypes were GCGT (lung cancer: 58.6%, control: 64.7%) and CCGG (case: 23.8%, control: 26.1%) (Table 4). Single omnibus test detected a significant association of these haplotype with lung cancer (p = 0.036). Further haplotype-specific test showed that haplotype GCGG increased lung cancer risk significantly (p = 0.015) when compared to the other haplotypes (Table 4). Similar analysis of leukemia patients did not reveal any evidence of association with leukemia (data not shown).

**Table 4:** Four marker haplotype frequencies and association with lung cancer.

No	Haplotype				Frequency		χ^2^	P value
	In.2…11827	In.3…11992	In.6…13494	In.7…14201	Case	Control		
1	G	C	G	T	0.586	0.647	0.696	0.404
2	C	C	G	G	0.238	0.261	0.124	0.725
3	G	C	G	G	0.063	0.003	5.884	**0.015**
4	C	C	A	T	0.000	0.028	2.130	0.145
5	C	A	G	T	0.095	0.028	3.650	0.056
6	C	C	G	T	0.019	0.032	0.317	0.573

These data together indicate that intron 7 SNPs 14181 and 14201 show an association with lung cancer risk in the Chinese female non-smoker population. Moreover, the haplotype analyses also indicate a correlation between the 4 tagSNP and increased lung cancer probability, highlighting that intronic SNPs may play a role in cancer susceptibility.

### Intron 7 SNPs and overall survival

**Figure 2:**
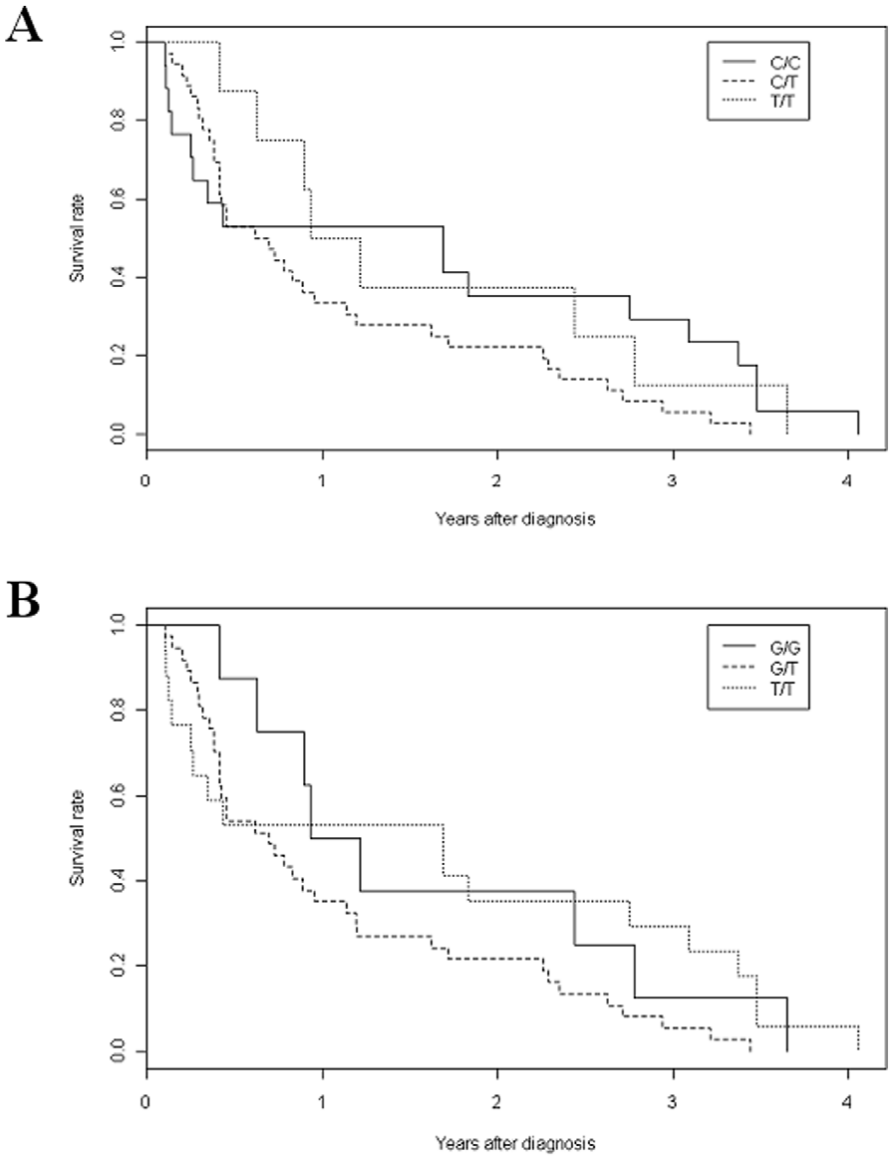
Kaplan-Meier curves of overall survival of lung cancer patients with different genotypes at intron 7, SNP14181 (A) or SNP14201 (B).

We therefore performed Kaplan-Meier analysis to determine if the different genotypes of the intron 7 SNPs would affect overall survival (OS). Median OS were found to be 1.69, 0.62 and 0.93 years for lung cancer patients with genotype C/C, C/T and T/T at SNP 14181, respectively (Figure 2A), while it was 0.93, 0.70 and 1.69 years for patients with genotypes G/G, G/T and T/T at SNP14201, respectively (Figure 2B). Log-rank tests did not detect any significant difference in OS among different genotypes at SNPs14181 (p = 0.112) and 14201 (p = 0.108). These data therefore indicate that although the intron 7 SNPs are associated with risk of lung cancer, there are no notable associations with OS in this limited study.

## Discussion

This work was undertaken to perform a detailed analysis of SNPs in the *p53* gene in the Chinese population, with the aim of uncovering new SNPs as well as to study previously noted SNPs in HapMap that have not been characterized in the Chinese cohorts [10]. Although many SNPs have been reported in the HapMap project, most contain only data from the US population (which comprises of 24 African Americans, 24 Asian Americans, 24 European Americans, 12 Hispanic American, 6 Native Americans) [10]. Our analysis confirmed the presence of SNPs noted in the HapMap project, and also uncovered several novel SNPs in introns 1 and 3. In addition, we focused our attention on other related and interesting SNPs, such as those in introns 2, 7 and 11, and have identified complete LD between the known codon 72 polymorphism (SNP12139) in exon 4 and the SNP11827 in intron 2, suggesting that they may be functionally related. Furthermore, we have noticed a strong correlation between the two linked SNPs in intron 7 with lung cancer predisposition.

When the allelic frequencies from our study was compared to the HapMap data on Chinese subjects, there was a general correlation, as evidenced by the exon 4 SNP12139, intron 6 SNP13494 and intron 7 SNP14201 (Table 1), validating this analysis. Based on this, further comparison of reported frequencies in the US population with the Chinese population revealed strong differences in two sets of SNPs; the intron 2 (11827)/exon 4 (12139) and the intron 7 SNPs (14181/14201). Essentially, there is an increase of the minor allele in the Chinese population compared to the US population in the above cases, similar to the originally described trend of an association of the exon 4 (codon 72) SNP with populations at different latitudes [27]. This therefore highlights that ethnicity is a critical factor in the distribution of allelic frequencies, and may be functionally relevant in determining p53 function.

The most striking observation was the identification of the linkage of the intron 2 SNP (11827) with the common codon 72 SNP in exon 4 (12139), which were in complete LD with each other. It was interesting that this was not noticed earlier, given the fact that the codon 72 SNP has been extensively studied with respect to cancer susceptibility. We have previously reported that the arginine or the proline variants of the exon 4 polymorphism, which arise due to the G or C nucleotides respectively, can be differentially expressed in healthy and cancer cases [17]. The regulation of the allele-specific differential expression is at present unclear. Thus, the recognition of the linkage between this SNP and of that in intron 2, especially with the C or G nucleotides co-segregating in both cases, may provide us with some clues to the regulation of the codon 72 SNP-specific expression, provided that the intron 2 SNP has transcription regulatory functions. This hypothesis is currently being tested. Moreover, the relevance of the intron 2 SNP has not been well characterized yet, especially with respect to cancer susceptibility, but given the recognition of complete LD with the exon 4 SNP, future work should shed light on its functional relevance. It is also noteworthy that the intervening SNPs in intron 3 are totally not linked to the intron 2 and codon 72 SNPs, suggesting rearrangements in the human *p53* gene have not affected this SNP pair, tempting us to speculate that this co-segregation may be important in regulating p53 functions.

We also attempted to compare the various SNPs with cancer susceptibility in a limited scale using samples from our previous studies on leukemia and female non-smoker lung cancer patients [32], [33]. Many of the SNPs characterized were not found to have any significant association with cancer risk. However, we noticed that the variant alleles of the intron 7 SNPs were associated with increased risk in the lung-cancer cohorts. Although these two SNPs were noted earlier, it was not recognized that they were completely linked [34]. Of these two SNPs, the SNP 14181 result in an *Apa1* restriction site when it is a T nucleotide, and one group has noted an association with risk of non-small cell lung cancer and oral neoplasm [35], [36]. However, no report has analyzed the association of SNP 14201 or both the intron 7 SNPs with cancer risk. Moreover, when we analyzed for association of tagSNPs with cancer susceptibility, we found that 4 tagSNPs in intron 2, 3, 6 and 7, with the GCGG haplotype increased lung cancer risk significantly. It is noteworthy that there is no significant LD among these 4 SNPs, but this haplotype combination, though infrequent in the population, was a useful indicator of cancer risk. Similar association was not apparent in our leukemia samples, and no correlation was noted with overall survival in this study, probably due to the sample size, or due to the differences in cancer type. Thus, further large-scale association studies would be useful in determining the relevance of all these SNPs with cancer susceptibility in various cancer types.

Finally, it is worth considering that many of the SNPs in the *p53* gene are in the intronic regions. Of these, at least those in introns 2, 6 and 7 show some association with cancer risk [11–14,35,36 and this study], suggesting that these changes may influence p53 functions. How these intronic regions affect cancer risk is unclear, but it is not inconceivable that they may contain promoter/enhancer activity that regulates p53 levels. Moreover, these regions may also regulate splicing of p53, resulting in the various splice variants that are emerging as important regulators of cell survival [37]. Hence, future work should shed light on the functional relevance of these intronic SNPs in regulating cancer risk.

## Materials and Methods

### Samples

Genomic DNA were prepared from peripheral blood as described by standard procedures using the Qiagen kit, from a total of 53 healthy individuals [17], and up to 44 leukemia patients [32] and up to 79 non-smoker lung-cancer patients [33], as have been described in our previous studies. Study was conducted informed written consent from participants with the approval of the National Cancer Centre Singapore and the Department of Hematology, Singapore General Hospital ethics committees and the Institutional Review Board of the National University of Singapore. The genomic DNA was used for genotyping and the data on overall survival was obtained from the hospital records with ethics approval.

Details of the leukemia and lung cancer group have been described in our previous studies aimed at determining the association between codon 72 polymorphism (exon 4 SNP) and cancer susceptibility in these populations [32], [33]. Essentially, all are Chinese Singaporeans, diagnosed at any one of three major hospitals in the country over the study period, and the demographic, smoking and other relevant information was obtained by in-person interview with a trained nurse, as described [32], [33].

### Genotyping and Sequencing

Genomic DNA was sequenced from nucleotide position 8634 at the 5′end of *p53* to nucleotide 19715 at the 3′ end of *p53*, covering exons 2 to 11 and parts of all intronic regions up to the 3′UTR. Primers used in the studies are detailed in Table 5. All 53 healthy samples were used for entire sequencing, and the indicated numbers of cancer samples available (as in Tables 2 and 3) were used for sequencing and genotyping. The new SNP information has been deposited in Genbank.

**Table 5:** Primers used and PCR conditions for sequencing *p53.*

Region covered	Forward Primer	Reverse Primer	PCR Conditions
In.1 to Ex.2	p53_8634F:5′-tgagctcttactgtgtgcc-3′	p53_10444R:5′-tgtgtggaccagcatcttg-3′	94°C, 3mins94°C, 30s55°C, 40s72°C, 30sRepeated for 30 cycles72°C, 5mins
	p53_9533F:5′-gcctgggcaacatagtga-3′	p53_10444R:5′-tgtgtggaccagcatcttg-3′	As for In.1 to Ex.2
	p53_10367F:5′-atggcagcctttgaaagc-3′	p53_11740R:5′-aggatctgactgcggctc-3′	As for In.1 to Ex.2
	p53_10922F:5′-gtgagacagttgttcttcc-3′	p53_11740R:5′-aggatctgactgcggctc-3′	As for In.1 to Ex.2
In.1 to In.4	p53_11584F:5′-tcagacactggcatggtgt-3′	p53_12403R:5′-aagcctaagggtgaagagga-3′	94°C, 3mins94°C, 30s53°C, 40s72°C, 30sRepeated for 30 cycles72°C, 5mins
In.4 to In.6	p53_12974F:5′-ttctttgctgccgtgttcca-3′	p53_13536R:5′-aggtcaaataagcagcagga-3′	As for In.1 to Ex.2
In.6 to In.9	p53_13894F:5′-acagagcgagattccatctc-3′	p53_14861R:5′-ctgatggcaaatgccccaat-3′	As for In.1 to Ex.2
In.9 to In.10	p53_17523F:5′-ctcaggtactgtgtatatac-3′	p53_17747R:5′-tggaatcctatggctttcca-3′	As for In.1 to Ex.2
In.10 to 3′UTR	p53_18303F:5′-agaggttgcggtgagctg-3′	p53_19715R:5′-cactctgggaggctgagac-3′	As for In.1 to Ex.2

### Statistical Analysis

The observed genotype frequencies of each SNP in the controls were tested for Hardy-Weinberg equilibrium (HWE) using Fisher's exact test. The frequency of minor allele at each SNP in HWE is reported together with 95% Confidence Interval (CI). P value on the basis of the 3 categorical genotypes, or allele variable, and a p trend value on the basis of the 3 level ordinal genotype variable (0: wildtype homozygotes, 1: heterozygotes, 2: mutant homozygotes) in the logistic regression model were calculated.

SNPs with minor allele frequency (MAF) of at least 1% among control sample were used to select tagSNPs based on pairwise r^2^ linkage disequilibrium (LD) map [38] using Tagger in Haploview version 4.0 (http://www.broad.mit.edu/mpg/haploview) [39], at a relatively stringent r^2^ threshold of 0.8. Haplotype analysis of these tagSNPs in this case-control study was carried out using PLINK (http://pngu.mgh.harvard.edu/purcell/plink/) [40]. For each haplotype with frequency at least 1% either in case or control, a single omnibus test was performed to test the effects of all these haplotypes jointly, and haplotype-specific test with 1 degree-of-freedom was also carried out for each haplotype using PLINK by comparing this specific haplotype with all others.

Kaplan-Meier estimated overall survival (OS) was carried out and log-rank test was used to compare the equivalence of OS among different genotypes at the two SNPs of intron 7. Kaplan-Meier estimated median OS were reported for each genotype at these two SNPs. All analyses were done using R version 2.7.1 (http://www.R-project.org).
